# Whole-Genome Sequence of *Aquamicrobium* sp. Strain SK-2, a Polychlorinated Biphenyl-Utilizing Bacterium Isolated from Sewage Sludge

**DOI:** 10.1128/genomeA.00439-15

**Published:** 2015-05-14

**Authors:** Young-Cheol Chang, Ken Sawada, Eun-Sook Kim, Kweon Jung, Shintaro Kikuchi

**Affiliations:** a Department of Applied Sciences, College of Environmental Technology, Muroran Institute of Technology, Muroran, Hokkaido, Japan; b Seoul Metropolitan Government Research Institute of Public Health and Environment, Gwacheon-si, Gyeonggi-do, South Korea

## Abstract

Here, we report the whole-genome sequence of *Aquamicrobium* sp. strain SK-2, a bacterium which can use 2,2′,4,4′,5,5′-hexachlorobiphenyl as the sole carbon source for its growth. An approximately 9.23-Mb genome sequence of SK-2 will greatly facilitate research efforts regarding the study of the polychlorinated biphenyl (PCB) degradation mechanism.

## GENOME ANNOUNCEMENT

Biphenyl and polychlorinated biphenyl (PCB) (a synthetic biphenyl) are persistent organic pollutants in the environment and are causing serious environmental problems around the world (1). In Japan, the manufacture, import, and use of PCBs were prohibited in 1974. However, environmental pollution with PCB is still ubiquitous in Japan. *Aquamicrobium* sp. strain SK-2 (accession no. AB612121) was originally isolated from sewage sludge in South Korea, and it can utilize PCB congeners containing one to six chlorine substituents, such as 2,2′,4,4′,5,5′-hexachlorobiphenyl (2). Strain SK-2 can also degrade biphenyl and PCB in the presence of high concentrations of NaCl and nitrate. In addition, the PCB degradation rate of strain SK-2 is higher than that of previously reported PCB congener-degrading bacteria (3, 4). Therefore, the isolated strain SK-2 might be a good candidate for the bioremediation of PCB-contaminated soil, especially in high-saline soils. Therefore, the whole-genome sequence of *Aquamicrobium* sp. SK-2 is reported here, and it can be useful for further investigating the roles of genes that are involved in biphenyl and PCB degradation.

We report the whole-genome sequence of *Aquamicrobium* sp. SK-2, the first whole-genome sequence of the genus *Aquamicrobium* and the first whole-genome sequence of the hexachlorobiphenyl-utilizing bacterium. Strain SK-2 was sequenced using a multiplex (8 samples on one lane) Illumina HiSeq 2000/2500 platform, yielding 29.8 million reads at 325× coverage. For the SK-2 datasets, we assembled the reads into a set of 180 contigs using Velvet (version 1.2.08) (5). The final circular genome of SK-2 has 9,230,439 bp, with an overall G+C content of 66%. Sequence annotation and open reading frame (ORF) prediction were performed using the Rapid Annotations using Subsystems Technology (RAST) version 2.0 pipeline (6). The rRNAs and tRNAs were identified using the search_for_RNAs script developed by Niels Larsen (6) and tRNAscan-SE (7), respectively. By these analyses, 96 tRNAs and 4 rRNA operons, comprising 5S, 16S, and 23S rRNA genes, were detected in the genome of SK-2.

Strain SK-2 has a genome (9.23 Mb) similar to those of other PCB degraders, such as *Burkholderia xenovorans* LB400 (9.73 Mb) (8), *Rhodococcus jostii* RHA1 (9.70 Mb) (9), and *Rhodococcus* sp. strain R04 (9.13 Mb) (10). The SK-2 whole-genome sequence may assist in the improvement of PCB bioremediation technology.

### Nucleotide sequence accession numbers. 

This whole-genome shotgun project has been deposited at DDBJ/EMBL/GenBank under the accession numbers BBWE01000001 to BBWE01000180 (BioProject no. PRJDB93210).
